# Field efficacy of a new mosaic long-lasting mosquito net (PermaNet^® ^3.0) against pyrethroid-resistant malaria vectors: a multi centre study in Western and Central Africa

**DOI:** 10.1186/1475-2875-9-113

**Published:** 2010-04-27

**Authors:** Vincent Corbel, Joseph Chabi, Roch K Dabiré, Josiane Etang, Philippe Nwane, Olivier Pigeon, Martin Akogbeto, Jean-Marc Hougard

**Affiliations:** 1 Institut de Recherche pour le Développement (IRD), UR016, Caractérisation et Contrôle des Populations de Vecteurs, 01 BP 4414 RP Cotonou, République du Bénin; 2 Centre de Recherche Entomologique de Cotonou (CREC), 06 BP 2604 Cotonou, République du Bénin; 3 Institut de Recherche en Sciences de la Santé (IRSS)/Centre Muraz (CM), 01 BP 390, Bobo-Dioulasso, Burkina Faso; 4 Organisation de Coordination pour la lutte contre les Endémies en Afrique Centrale (OCEAC), BP. 288, Yaoundé, Cameroon; 5 Walloon Agricultural Research Centre (CRA-W), Agriculture and Natural Environment Department, Plant Protection Products and Biocides Physico-chemistry and Residues Unit, Rue du Bordia, 11 B-5030, Gembloux, Belgium; 6 Institut de Recherche pour le Développement (IRD), BP 1386 Dakar, République du Sénégal

## Abstract

**Background:**

Due to the spread of pyrethroid-resistance in malaria vectors in Africa, new strategies and tools are urgently needed to better control malaria transmission. The aim of this study was to evaluate the performances of a new mosaic long-lasting insecticidal net (LLIN), i.e. PermaNet^® ^3.0, against wild pyrethroid-resistant *Anopheles gambiae s.l*. in West and Central Africa.

**Methods:**

A multi centre experimental hut trial was conducted in Malanville (Benin), Vallée du Kou (Burkina Faso) and Pitoa (Cameroon) to investigate the exophily, blood feeding inhibition and mortality induced by PermaNet^® ^3.0 (i.e. a mosaic net containing piperonyl butoxide and deltamethrin on the roof) comparatively to the WHO recommended PermaNet^® ^2.0 (unwashed and washed 20-times) and a conventionally deltamethrin-treated net (CTN).

**Results:**

The personal protection and insecticidal activity of PermaNet 3.0 and PermaNet^® ^2.0 were excellent (>80%) in the "pyrethroid-tolerant" area of Malanville. In the pyrethroid-resistance areas of Pitoa (metabolic resistance) and Vallée du Kou (presence of the L1014F *kdr *mutation), PermaNet^® ^3.0 showed equal or better performances than PermaNet^® ^2.0. It should be noted however that the deltamethrin content on PermaNet^® ^3.0 was up to twice higher than that of PermaNet^® ^2.0. Significant reduction of efficacy of both LLIN was noted after 20 washes although PermaNet^® ^3.0 still fulfilled the WHO requirement for LLIN.

**Conclusion:**

The use of combination nets for malaria control offers promising prospects. However, further investigations are needed to demonstrate the benefits of using PermaNet^® ^3.0 for the control of pyrethroid resistant mosquito populations in Africa.

## Background

Malaria remains a major public health problem. Last global estimates of the malaria disease burden in 2006 indicate that at least 250 million clinical cases occurred each year, with around 1 million deaths of which 90% occurred in sub-Saharan Africa [1,2]. Recommendations of the World Health Organization (WHO-Roll Back Malaria programme) to combat malaria include artemisinin-based combination therapy (ACT) and long-lasting insecticidal nets (LLIN), supported by indoor residual spraying of insecticide (IRS) and intermittent preventive treatment in pregnancy (IPT) [2]. Recent deployment of such strategies has showed important reduction in malaria-associated morbidity and mortality in settings with moderate to high transmission levels in sub-Saharan Africa [3-5]. Eight LLINs are now recommended by WHOPES for malaria control [6]. All of them contain pyrethroids because of their fast and high insecticidal properties on mosquitoes as well as their low mammalian toxicity [7].

Unfortunately, pyrethroid resistance is now widespread in malaria vectors including Western [8], Central [9], Eastern [10], and Southern Africa [11,12]. Resistance mechanisms are divided into two groups: metabolic (i.e. alterations in the levels or activities of detoxification proteins) and target site (i.e. non-silent point mutations within structural receptor genes) [13]. Mutations (L1014F or L1014S) on the gene encoding for the sodium channel, known as knockdown resistance (*kdr*), cause resistance to DDT and/or pyrethroid insecticides [14,15]. Over-expression of enzymes related to insecticide resistance involves the cytochrome P450-dependent monooxygenases (P450), the carboxylesterases (COE), and the glutathione-S transferases (GST) [16]. Among these three families, P450s can play a primary role in pyrethroid detoxification and resistance in malaria vectors as recently shown in Benin [17], Cameroon [18], Ghana [19] and South Africa [20].

There are more and more evidences in the recent literature to support that pyrethroid resistance may seriously impact on malaria vector control [21]. An experimental hut study carried out in southern Benin in 2004 (Ladji) showed a rather low insecticidal effect of permethrin-treated nets, at WHO recommended dosages against *Anopheles gambiae *[22]. A recent study carried out in the same locality with lambda-cyalothrin used for ITNs and IRS showed a major loss of efficacy associated with *kdr *resistance [23]. Reduced efficacy of permethrin-impregnated bed nets against *An. gambiae *strain sharing oxidase-based pyrethroid tolerance was also reported in Cameroon [24] and Kenya [25,26]. Moreover, an increasing number of countries (such as Benin, Ghana and Nigeria) reported the co-occurrence of the L1014F *kdr *mutation and increased levels of P450s within the same Anopheline populations [17,19]. As demonstrated in *Culex quinquefasciatus*, multiplicative interaction (epistasis) between these two types of resistance can lead to extremely high level of resistance to pyrethroids [27,28]. Thus, the challenge is not only to manage and control pyrethroid-resistant mosquitoes, but also to deal with the development of "multiple resistance" that may confer resistance to all insecticide classes used in public health (i.e. DDT, carbamates, etc.). Innovative tools are then urgently needed to ensure more effective control of resistant malaria vectors and to help developing countries to achieve the malaria-related Millennium Development Goals i.e. 75% reduction of malaria burden until 2015 [2].

Among the new tools available in public health, PermaNet^® ^3.0, has been designed to improving efficacy against pyrethroid-resistant mosquito populations [29]. PermaNet^® ^3.0 is a mosaic net combining deltamethrin-coated-polyester side panels and a deltamethrin plus piperonyl butoxide (PBO) incorporated-polyethylene roof. PBO has been incorporated to the net as it showed to enhance the effects of deltamethrin against insects by inhibiting metabolic defence systems, mainly P450s [30].

In this paper, a multi centre study was carried out in western and central Africa to evaluate the performances of this new LLIN technology (PermaNet^® ^3.0) in comparison with the classical PermaNet^® ^2.0 recommended by WHO. Experimental hut trials were conducted in Malanville (Benin), Pitoa (Cameroon) and Vallée du Kou (Burkina Faso), where *An. gambiae *populations showed different levels and types of pyrethroid resistance (i.e. metabolic *versus *target site modification). Standard WHO procedures in phase II were followed to investigate the efficacy of unwashed and 20 times washed LLINs in terms of induced exophily, blood-feeding inhibition and mortality.

## Methods

### Study areas

**Figure 1:**
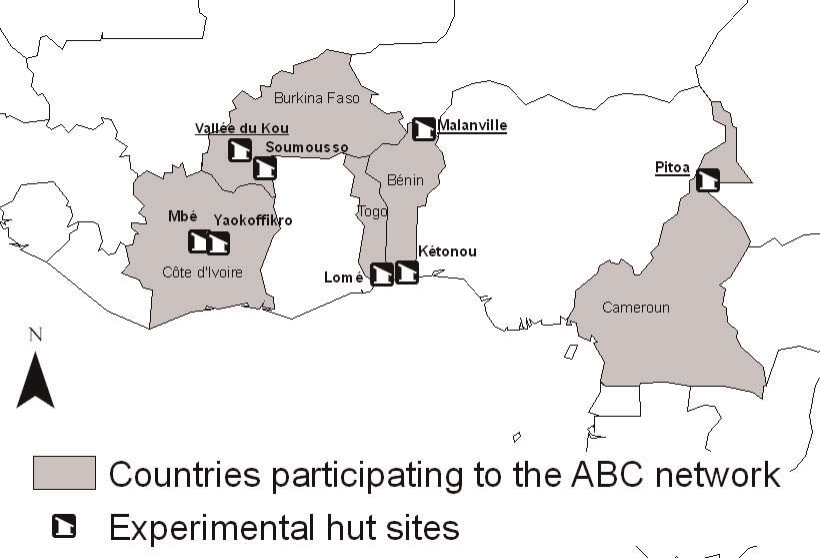
**Experimental hut stations belonging to the Anopheles Biology & Control (ABC) network**. Huts from Malanville (Northern Benin), Pitoa (Northern Cameroon), and Bobo-Dioulasso (Southern Burkina Faso) were used in this study.

This study was conducted in three experimental stations belonging to the Anopheles Biology & Control (ABC) network (Figure 1). Two stations are located in western Africa whereas the third one is located in Central Africa. Each site presents different pattern of pyrethroid-resistance among *An. gambiae *s.l. populations.

Malanville (11°87N; 03°38E) is in northern Benin, 800 km from Cotonou, in an irrigated rice-growing valley. The climate is tropical soudanian, characterized by a dry season from December to June and a rainy season from July to November. *Anopheles gambiae s.s*. M cytotype is the main malaria vector in this area and presents very low levels of pyrethroid resistance [31].

Pitoa (9°21N; 13°31E) is a small village, with around 5,000 inhabitants, located at 15 km from Garoua, in an area of extensive cotton cultivation in Northern Cameroon (around 35 000 ha cultivated). *Anopheles gambiae s.l*. and *Anopheles funestus s.l*. are the main malaria vectors in this area. *Anopheles arabiensis *is predominant and showed moderate level of resistance to permethrin, deltamethrin and DDT [32] due to increased oxidase and esterase activity [18,33].

Vallée du Kou is a large rice-growing area (1,200 ha), located at 30 km Northern Bobo-Dioulasso and comprised seven villages, surrounded by wooded savannah. VK7 (11°24N; 4°24E) is a village located on the outskirts of rice fields. Both M & S molecular forms of *An. gambiae *co-exist in sympatry but the M form is mostly present during the dry season [34]. *Anopheles gambiae *showed high level of resistance to pyrethroids due to the presence of the *Kdr *mutation occurring at high allelic frequency among the molecular M and S forms [35].

### Determination of the pyrethroid resistance status of *An. gambiae s.l*

In each site (Malanville, Pitoa and Vallée du Kou) and just prior to the trials, resistance of *An. gambiae s.l*. to pyrethroids was checked using WHO cylinder test [36]. Four batches of 25 unfed females, aged 2-5 days, were exposed to deltamethrin-impregnated papers for 1 h (0.05%) and held to observe mortality after 24 h, then stored at 4°C for further molecular studies. A sub-sample of 30 mosquitoes per locality was identified to sibling species and for the relative frequency of the molecular M & S forms using standard PCR methods [37,38]. The method of Martinez-Torrez *et al *[14] was used for the molecular detection of the L1014F *kdr *mutation.

### Study design and experimental huts

Experimental huts are specially designed to test vector control products against freely entering mosquitoes under natural but controlled conditions [36]. The 3.5 × 2 × 2 m huts were built with local materials and designed with four entry baffles that enabled mosquitoes to fly into the hut but then hindered their escape from the hut. A veranda trap made of polyethylene sheeting and mesh screening (2 m long × 1.5 m wide × 1.5 m high) projected from the back wall of each hut. Movement of mosquitoes between the huts and the verandas was unimpeded during the night. Each hut rested on a concrete base surrounded by a water-filled moat to prevent entry of ants that would otherwise eat mosquitoes knocked down on the floor of the hut.

In each country, the treatments were randomly allocated to six experimental huts, which did not differ between them for their mosquito attractiveness in absence of treatment. Adult volunteers have been recruited among the inhabitants of the villages where experimental huts were implemented. They have been informed on the objective of this study and signed (or through a literate witness if illiterate) an informed consent. They entered the hut at dusk (7:00pm) and remained inside until dawn (5:30 am) of the next morning. Early in the morning, dead mosquitoes were collected from the floor of the hut as well as from the exit traps and inside the nets; resting mosquitoes were collected using aspirators from inside the net and from the walls and roof of the hut and exit traps. Mosquitoes were scored by location as dead or alive and as fed or unfed. Live mosquitoes were placed in small cups and provided with access to sugar solution for 24 hours to assess the delayed mortality. To minimize bias related to mosquito attractiveness of each volunteer and spatial variation in mosquito densities, the volunteers and bed nets were rotated between huts each day according to a Latin square design [36].

Efficacy of each treated arms was expressed in terms of deterency, induced exophily, blood-feeding inhibition, and mortality. This multi-centre trial included the determination of the efficacy of unwashed and 20 times washed PermaNet^® ^3.0 comparatively to the WHO recommended PermaNet^® ^2.0 and a conventional deltamethrin-treated net washed to just before exhaustion (as defined by WHOPES guidelines [33]). Their impact on the behaviour of wild pyrethroid resistant *An. gambiae s.l*. and *An. arabiensis *mosquitoes was also evaluated.

### Mosquito net treatments

In each country, six treated arms were randomly allocated to huts:

1. Untreated net (same fabric - polyester on the side with a strengthened 70 cm lower border/polyethylene on top)

2. PermaNet^® ^3.0 unwashed

3. PermaNet^® ^2.0. unwashed

4. PermaNet^® ^3.0 washed 20 times

5. PermaNet^® ^2.0 washed 20 times

6. Polyester net conventionally treated with deltamethrin at 25 mg a.i./m^2 ^and washed to just before exhaustion i.e. 95% Knock down after 1 h of contact and/or 80% mortality after 24 h [36].

The LLINs (PermaNet^® ^2.0 and PermaNet^® ^3.0) were provided by Vestergaard Frandsen SA, (Switzerland). PermaNet^® ^2.0 is a deltamethrin-coated LN, made of knitted multi-filament polyester fibres and is treated with deltamethrin at a target concentration of 55 mg/m^2 ^(= 1.8 g/kg for a 75-denier net used in Malanville and Pitoa and = 1.4 g/kg for a 100-denier net in Vallée du kou). PermaNet^® ^2.0 received WHOPES full recommendation for LLIN in 2009. PermaNet^® ^3.0 product is a combination of different long-lasting technologies. The roof of PermaNet^® ^3.0 utilizes deltamethrin and PBO incorporated into monofilament polyethylene yarn of 100 denier (warp-knitted fabric, with weight of 40 ± 15% g/m^2^) at the target dosage of 4.0 g AI/kg and 25 g AI/kg of netting material, respectively. The side panels of PermaNet^® ^3.0 are made of multi-filament polyester fibres, treated with deltamethrin in a resin coating (75 denier, warp-knitted fabric, atlas construction). The side netting has two parts: a strengthened lower part, so-called border (70 cm) by using 75 denier yarn (weight 40 ± 10% g/m^2^) and a side panel made of 75 denier (weight of 30 ± 10% g/m^2^). The target dose of deltamethrin in the side panels is 2.8 g AI/kg of netting material, i.e. 115 mg AI/m^2 ^of the border and 85 mg AI/m^2 ^of the remaining of the side panels.

The polyester net was conventionally treated with deltamethrin at 25 mg AI/m^2 ^and washed to just before the point of exhaustion (i.e. <80% mortality or <95% knock down). This treatment was used as a positive control. Each net was deliberately holed with six holes (4 cm × 4 cm) to simulate a torn net [36].

### Residual activity and wash resistance of the net treatments

The bio-efficacy of each treatment was determined before washing and after field testing by exposing 2 to 5 days old unfed females of the susceptible *An. gambiae *Kisumu strain in WHO cone bioassays [36]. This test consists to expose female mosquitoes to each part of the nets for 3 min and to measure the knock down time after 60 minutes and the mortality after 24 H. A mean of 50 mosquitoes was tested per net and results pooled for analysis. Sugar solution was provided during the 24-h holding period, and the temperature was kept at around 25°C. The standardized WHO protocol was used for washing the nets [36].

### Chemical analysis

Determination of deltamethrin and PBO content on nets, before washing and after the field testing was investigated using a new method developed by the WHO Collaborating Centre for the Quality Control of Pesticides (Walloon Agricultural Research Centre, Gembloux, Belgium)[39]. In each country, five pieces of netting (about 30 cm × 30 cm) were cut from the roof and side panel and stored in aluminium foil for subsequent chemical analysis. The side panels and roof were tested separately for the PermaNet^® ^3.0. The chromatographic determination of deltamethrin, deltamethrin R-isomer and piperonyl butoxide was performed by gas chromatography with flame ionization detection (GC-FID) after extraction by refluxing with xylene. Before the analysis of samples, the analytical method was successfully validated for its specificity, linearity of detector response, accuracy, repeatability and reproducibility.

### Statistical analysis

Data from *in situ *bioassays were compared between each net using a Chi square test at 95% confidence interval, using the Minitab software version 12.2. In each study site, the number of mosquitoes of each species entering the huts was compared by species and analysed using the non parametric Kruskal-Wallis test. The proportion of mosquitoes that exited early (induced exophily), the proportion that was killed within the hut (mortality) and the proportion that successfully blood fed (blood feeding rate) were compared and analysed using the logistic regression (Addinsoft, 2009, XLSTAT 2006). The percentage personal protection (PP) was calculated as (BFC-BFT)/(BFC) * 100, where BFC is the total number of blood fed females in the control hut and BFT the total number of blood-fed female mosquitoes in the treated hut. The overall killing effect (KE) of a treatment was calculated as (DT-DC)/(TC) * 100, where DT is the total number of dead mosquitoes in the treated hut, DC the total number of dead mosquitoes in the control hut and TC is the total number of mosquitoes collected in the control hut [36].

## Results

### Vector population and pyrethroid resistance

Table 1 summarizes the sibling species, molecular forms and pyrethroid resistance status of *An. gambiae s.l*. collected in the three experimental hut stations. *Anopheles gambiae s.s*. was predominant in Malanville (95%) and Vallée du Kou (100%), whereas *An. arabiensis *was predominant (95%) in Pitoa. The composition of *An. gambiae s.s*. was 100% M form for the Malanville sample and 80%/20% S/M molecular forms for the Kou Valley sample. Different levels of deltamethrin resistance were reported in the three study sites; the most "susceptible" population was found in malanville (i.e. 85% mortality to deltamethrin), the most resistant in Vallée du Kou (23% mortality) and the population of Pitoa being intermediate (70% mortality). The *kdr *mutation was present at very high frequency (>80%) in both molecular M & S forms in Vallée du Kou whereas it was only 16% in the M form in Malanville. In Pitoa, the *kdr *mutation was almost absent (<5%) and deltamethrin resistance in *An. arabiensis *was associated with elevated esterase and oxidase activities as described previously [18,33].

**Table 1:** Species, molecular forms and pyrethroid resistance status of *An. gambiae s*.l in the three experimental hut stations.

Country	Species		Molecular form *		Kdr frequency		Mortality % **	Resistance status ***
	***An. gambiae *** ***s.s***.	***An. arabiensis***						
							
**Malanville** **(Benin)**	95%	5%	100% M		16%		85%	**Resistance ** **suspected** (kdr mutation, oxidase)
**Pitoa** **(Cameroon)**	5%	95%	100% S		<5%		70%	**Resistance** (oxidase +Esterase)
**Vallée du kou** **(Burkina Faso)**	100%	0%	15% M	85% S	>80 % M	>80 % S	23%	**Resistance** (kdr mutation)

*An. gambiae s.s.  **deltaméthrine 0.05%, *** from [30 and][17]

### Insecticide residual activity

With conventionally deltamethrin-treated nets (CTN), KD and mortality decreased below the WHO threshold (95% and 80% respectively) after four washes (respectively 73% and 71%). Hence, three washes were considered as the number of washes required before exhaustion. Residual activity of PermaNet^® ^2.0 and PermaNet^® ^3.0 as measured by WHO cone bioassay tests showed no significant decrease in efficacy after washing and/or field testing (Table 2).

**Table 2:** Residual activity (as determined by WHO cone bioassays on susceptible Kisumu strain) of unwashed and washed Permanet 2.0 and Permanet 3.0 in comparison with Conventionally Treated nets (CTN) washed to just before exhaustion in the three experimental hut stations.

Country	Conditions	Untreated net	Permanet 2.0 unwashed	Permanet 2.0 20× washed	Permanet 3.0 unwashed	Permanet 3.0 20× washed	CTN*
	Before washing	0.0^a ^(52)	100^b ^(52)	100^b ^(53)	100^b ^(54)	100^b ^(53)	100^b ^(53)
**Malanville** **(Benin)**	After washing or field testing	0.0^a ^(56)	100^b ^(63)	97^b ^(62)	100^b ^(61)	100^b ^(59)	89^c ^(64)
	Before washing	0.0^a ^(52)	100^b ^(55)	100^b ^(55)	100^b ^(50)	100^b ^(56)	100^b ^(54)
**Pitoa** **(Cameroon)**	After washing or field testing	3.6^a ^(56)	100^b ^(63)	98^b ^(61)	100^b ^(65)	100^b ^(60)	81^c ^(63)
	Before washing	0.0^a ^(56)	100^b ^(55)	100^b ^(57)	100^b ^(59)	100^b ^(59)	100^b ^(58)
**Vallée du Kou** **(Burkina Faso)**	After washing or field testing	0.0^a ^(62)	100^b ^(63)	98^b ^(59)	100^b ^(55)	100^b ^(54)	95^b ^(57)

* Three washes were required to reach the point to just before exhaustion.

Two Values in the same raw sharing same letter do not significantly differ (P > 0.05).

Values in bold represent the number of tested mosquitoes per treatment.

### Efficacy of treatments in experimental huts

The experimental hut trials were conducted from September till November 2007 in the Vallée du Kou and from July till September 2008 in Malanville and Pitoa. Thirty-six night collections (one Latin square) were required in Vallée du Kou and Pitoa to collect sufficient number of Anopheline mosquitoes for statistical analysis, whereas 72 nights collection (two Latin squares) were required in Malanville to obtain a correct density. In overall, 1,594 *An. gambiae s.l*. mosquitoes were collected in the control (untreated) huts among which 908 (equivalent 19 Anopheles bites per man per night), 401 (eq. 5.8 bites per man per nigh) and 285 (eq.1.5 bites per man per man) were found in Vallée du Kou, Pitoa and Malanville, respectively.

#### Deterency

A significant reduction in entry rates (deterrency) was noted with the unwashed PermaNet^® ^2.0 and 3.0 in Vallée du Kou and Pitoa compared to the untreated (control) arm whereas no significant reduction was noted in Malanville regardless the treatments (P < 0.05, see Additional file 1).

#### Induced exophily

**Figure 2:**
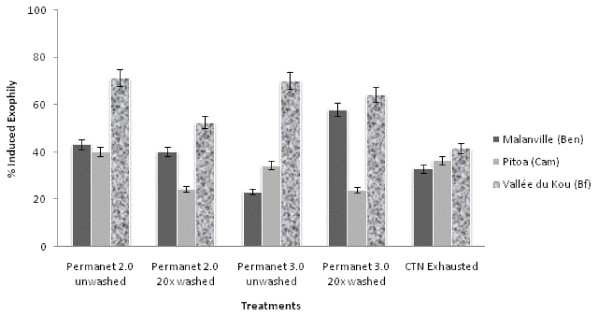
**Comparison of Induced Exophily obtained for unwashed and washed PermaNet^® ^2.0, PermaNet^® ^3.0 and CTN against free flying *An.gambiae s.l*. in experimental huts**.

In the control huts, the exophily in Pitoa did not differ significantly from the two others study sites, but the exophily rate in Malanville was significantly higher than in Vallée du Kou (p = 0.018) (see Additional file 1). The exophily induced by each treated hut is illustrated in Figure 2 and summarized in Additional file 2. The proportion of mosquitoes found in the veranda trap with PermaNet^® ^2.0 and 3.0 (washed or unwashed) was greater in Vallée du Kou (from 67 to 80%) than in Pitoa and Malanville (from 51 to 67%). Both LLINs (washed or unwashed) induced significantly more exophily than the untreated nets, regardless of the ecological settings. PermaNet^® ^3.0 (washed or unwashed) did not induce significantly higher exophily than PermaNet^® ^2.0 (washed or unwashed), except in Vallée du Kou where the proportion of mosquitoes found in the veranda trap was higher with PermaNet^® ^3.0 washed 20 times (75%) than PermaNet^® ^2.0 washed 20 times (67%) (P < 0.05).

#### BFI and personal protection

The proportion of mosquitoes that succeeded to take a blood meal with untreated holed nets was significantly lower from the area of Malanville (38%), to Pitoa (52% of blood fed females caught; p = 0.0205) and Vallée du Kou (75% of blood fed females caught; p = 0.0002) (see Additional file 2).

**Figure 3:**
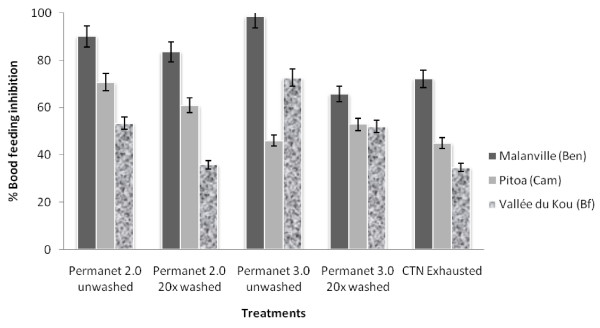
**Comparison of Blood Feeding Inhibition (BFI) rates obtained for unwashed and washed PermaNet^® ^2.0, PermaNet^® ^3.0 and CTN against free flying *An.gambiae s.l*. in experimental huts**.

The blood feeding inhibition (BFI) rates induced by each treated hut are illustrated in the Figure 3 and summarized in Additional file 2. The proportion of mosquitoes that succeeded to take a blood meal in the treated huts differed according to the study site; the BFI ranged from 65 to 98% in Malanville, from 45 to 71% in Pitoa and from 34 to 72% in Vallée du Kou. In Malanville, the BFI of PermaNet^® ^3.0 washed 20 times (65%) was significantly lower than for Permanet^® ^3.0 unwashed (98%) and PermaNet^® ^2.0 unwashed or washed 20 times (respectively 90% and 84%). In Pitoa, PermaNet^® ^2.0 induced a higher BFI, although this was only significant for unwashed nets. In contrast, BFI was higher with PermaNet^® ^3.0 over PermaNet^® ^2.0 in Vallée du Kou for both unwashed and washed bed nets (P < 0.05).

The personal protection of PermaNet^® ^2.0 and 3.0 was good when the LLIN was unwashed (from 80% in Pitoa to 99% in Malanville) but much lower when the nets were washed 20 times, especially in the pyrethroid resistance area of Vallée du Kou (44% and 62% for PermaNet^® ^2.0 and PermaNet^® ^3.0 respectively). In this resistance area of Vallée du Kou, PermaNet^® ^3.0 conferred a significantly better protection than the PermaNet^® ^2.0 (p = 0.0006 for unwashed LLINs; p = 0.0024 for washed LLINs).

#### Insecticidal activity

Mortality of mosquitoes in the control huts was low (around 5%) in Malanville and Vallée du Kou where *An. gambiae s.s*. was predominant. In contrast, higher mortality (13%) was recorded in Pitoa where *An. arabiensis *was found in higher proportion.

**Figure 4:**
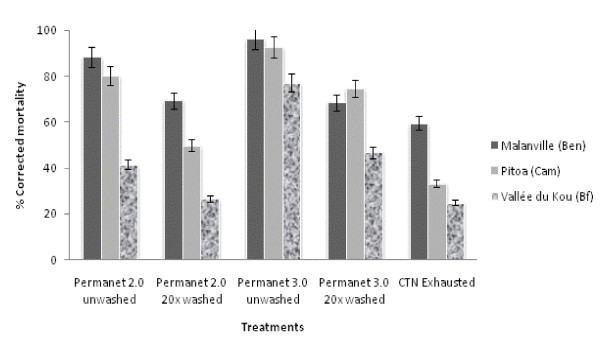
**Comparison of corrected mortality rates obtained for unwashed and washed PermaNet^® ^2.0, PermaNet^® ^3.0 and CTN against free flying *An.gambiae s.l*. in experimental huts**.

The mortality induced by each treated arm is illustrated in Figure 4 and summarized in Additional file 3. As for the blood feeding behaviour, the proportion of *An. gambiae *killed by the treated nets greatly differed according to the entomological setting. The corrected mortality was high in Malanville (from 61% for CTN to 96% for PermaNet^® ^3.0) and, in a lesser extent, Pitoa (from 41% for CTN to 93% for PermaNet^® ^3.0). In contrast, the mortality in Vallée du Kou ranged from 28% for CTN to 78% for PermaNet^® ^3.0. Regarding the LLINs only, mortality (~69%) was similar between PermaNet^® ^3.0 and PermaNet^® ^2.0 washed 20 times in Malanville whereas in Pitoa and Vallée du Kou PermaNet^® ^3.0 (washed or unwashed) induced significantly more mortality than PermaNet^® ^2.0 (p < 0.05). In all settings, washing the nets 20 times significantly reduced the number of mosquitoes being killed by the LLINs (p < 0.05).

The overall insecticidal effects of unwashed PermaNet^® ^2.0 and PermaNet^® ^3.0 were high in Malanville and Pitoa (from 80 to 96%) comparatively to Vallée du Kou (from 41 to 77%). This trend was stronger with washed LLINs especially in Vallée du Kou where the insecticidal activity of PermaNet^® ^3.0 and PermaNet^® ^2.0 decreased to 46% and 26%, respectively (see Additional file 3). The side effect questionnaires collected during the field trials did not reveal any adverse effects (symptoms or troubles) related to the use of any treated arms.

### Deltamethrin and PBO content on mosquito nets

In the three study sites, neither deltamethrin nor PBO was detected (limit of quantification = 0.01 g/kg for deltamethrin and 0.1 g/kg for PBO) in the untreated nets, hence confirming that no contamination occurred during the rotations of the nets (Table 3). The active ingredient content for unwashed PermaNet^® ^3.0 (deltamethrin and PBO) and PermaNet^® ^2.0 (deltamethrin) complied with the target doses (± 25%), except for one PermaNet^® ^2.0 (Pitoa) for which the average deltamethrin content on the side panels (2.53 g AI/kg) was just above the upper limit (2.25 g AI/kg) of the target dose. No major loss of deltamethrin and/or PBO content (from nil to 17%) was reported for unwashed PermaNet^® ^2.0 (deltamethrin) and PermaNet^® ^3.0 (deltamethrin and PBO) after the field testing. However, the loss of deltamethrin after 20 washes was relatively important for both PermaNet^® ^2.0 (from 59 to 85%) and PermaNet^® ^3.0 in the side panels (from 71 to 87%). However, the deltamethrin and PBO content in the roof panel remained high (>60% retention) in PermaNet^® ^3.0 after 20 washes. The deltamethrin content for unwashed CTN (0.8 g AI/kg) complied with the initial target doses (± 25%). After 3 washes the loss of active ingredient ranged from 85 to 91%.

**Table 3:** Determination of deltamethrin and PBO content on mosquito nets in the three experimental hut trials.

Country	Treatment	Target dose*	Deltamethrin/PBO content (g/kg)		Loss of active ingredient (%)
		**g/kg (IC95)**	**Before washing** **and testing**	**After testing**	**g/kg (IC95)**
		
**Malanville** **(Benin)**	Untreated net	0	<0.01	<0.01	
	Permanet 2.0	1.8 [1.35-2.25]	2.09	1.74	17%
	Permanet 2.0 20×	1.8 [1.35-2.25]	**2.41**	0.99	59%
	Permanet 3.0	2.8 [2.1-3.5]	2.61	2.32	11%
	Permanet 3.0 Roof	4 [3-5]	3.69	3.00	19%
	Permanet 3.0 20×	2.8 [2.1-3.5]	2.58	0.53	79%
	Permanet 3.0 roof 20×	4 [3-5]	3.69	3.16	14%
	CTN exhausted	0.8 [0.6-1.0]	0.59	0.09	85%
	PBO Permanet 3.0	25 [18.75-31.25]	20.8	23.1	+11%
	PBO Permanet 3.0 20×	26 [18.75-31.25]	20.7	12.4	40%
	
**Pitoa** **(Cameroon)**	Untreated net	0	<0.01	<0.01	
	Permanet 2.0	1.8 [1.35-2.25]	**2.53**	2.51	1%
	Permanet 2.0 20×	1.8 [1.35-2.25]	**2.65**	0.41	85%
	Permanet 3.0	2.8 [2.1-3.5]	2.44	2.50	-3%
	Permanet 3.0 Roof	4 [3-5]	3.21	3.22	0%
	Permanet 3.0 20×	2.8 [2.1-3.5]	2.48	0.33	87%
	Permanet 3.0 roof 20×	4 [3-5]	3.38	2.57	24%
	CTN exhausted	0.8 [0.6-1.0]	0.88	0.08	91%
	PBO Permanet 3.0	25 [18.75-31.25]	26.0	29.8	+15%
	PBO Permanet 3.0 20×	26 [18.75-31.25]	28.0	17.7	37%
	
**Vallée du kou** **(Burkina Faso)**	Untreated net	0	<0.01	<0.01	
	Permanet 2.0	1.4 [1.05-1.75]	1.31	1.32	-1%
	Permanet 2.0 20×	1.4 [1.05-1.75]	1.30	0.27	79%
	Permanet 3.0	2.8 [2.1-3.5]	2.89	2.94	+2%
	Permanet 3.0 Roof	4 [3-5]	4.33	4.07	6%
	Permanet 3.0 20×	2.8 [2.1-3.5]	3.10	0.90	71%
	Permanet 3.0 roof 20×	4 [3-5]	4.18	3.36	20%
	CTN exhausted	0.8 [0.6-1.0]	0.72	0.11	85%
	PBO Permanet 3.0	25 [18.75-31.25]	23.1	20.4	12%
	PBO Permanet 3.0 20×	26 [18.75-31.25]	22.9	15.1	34%

* Target dose for Permanet 2.0 was 1.8 g/kg ± 25% for 75 denier's nets in Benin and Cameroon and 1.4 g/kg ± 25% for 100 deniers net in Burkina Faso

## Discussion

A multi-centre experimental hut study was carried out to assess the efficacy of PermaNet^® ^3.0 against wild pyrethroid-tolerant *An. gambiae s.s*. (Malanville), *kdr*-resistant *An. gambiae s.s*. (Vallée du Kou) and pyrethroid-resistant *An. arabiensis s.l*. showing metabolic resistance (Pitoa). The ABC network offers ideal conditions to address this objective based on the existence of eight experimental hut stations in different ecological and entomological settings (Figure 1). In the present study, three stations where *An. gambiae s.l*. showed different level and type of resistance to pyrethroids were selected to assess whether PermaNet^® ^3.0 may represent a more potent technology than PermaNet^® ^2.0 against pyrethroid resistant mosquito populations.

### Differences in behavioural responses between wild Anopheline populations

The results from the control huts showed that the behavioural preferences of Anopheline populations (in terms of endophily/exphily) significantly differ between the three sites as expected from literature on trophic behaviour of malaria vectors [40-42]. It confirms that the behaviour of Anopheline populations depend on several factors including the species, the molecular forms, the resistance mechanisms and other environmental variables [40-42]. Interestingly, there is a difference in mortality rate in the control huts between *An. arabiensis *collected in Pitoa (12%) and the two others *An. gambiae *populations from Malanville and Vallée du Kou (<5%). Unfortunately, this study did not allow to decipher on the causes of this difference of mortality (behavioural preference, environmental conditions, etc.) but other authors have already reported similar mortality rates of *An. arabiensis *(10%) in experimental huts [43]. Nevertheless these differences shed light on the need for further investigations on behavioural preferences of wild populations of *An. gambiae s.s*. and *An. arabiensis*.

### Comparison between PermaNet^® ^2.0 and 3.0

The chemical analysis confirmed that in overall, unwashed nets were impregnated with the appropriate target dose of deltamethrin and PBO. Although efficacy of 20 times washed PermaNet^® ^3.0 and PermaNet^® ^2.0 was good, a rather high loss of insecticide was noted in the side panels (Table 3). Nevertheless, the deltamethrin and PBO retention in the roof was around 2.5 times higher than that of deltamethrin in the side panels, showing that the retention is better with incorporated polyethylene than with coated polyester [39].

This study first demonstrated a better or equal impact of PermaNet^® ^3.0 washed 20 times on mortality and blood feeding inhibition of major malaria vectors compared with that of the conventionally treated polyester nets (25 mg/m^2 ^AI) washed until just before exhaustion. This confirms that the PermaNet^® ^3.0 fulfils the WHOPES efficacy criteria of Phase II studies for LLIN.

Regarding the two LLINs, unwashed Permanet 3.0 induced significantly higher BFI and mortality than Permanet 2.0 in Vallée du Kou and Malanville. In the locality of Pitoa, the BFI was however higher with Permanet 2.0 than Permanet 3.0 but the mortality was still higher with Permanet 3.0. After 20 washes, the PermaNet^® ^3.0 also induced higher insecticidal effect than PermaNet^® ^2.0 in the pyrethroid resistance areas of Pitoa and Vallée du Kou, but performed equally in the area of Malanville.

One should note that in areas with high resistance levels (Vallée du Kou) 50% of resistant mosquitoes survived after exposure to PermaNet^® ^3.0 relative to 75% survival after exposure to PermaNet^® ^2.0. It remains to be seen if the gain of efficacy of PermaNet^® ^3.0 over PermaNet^® ^2.0 is enough to control highly pyrethroid-resistant malaria vector populations. Here, it is difficult to conclude on the benefit of using PBO on the roof because the deltamethrin content on PermaNet^® ^3.0 was approximately twice higher than that of PermaNet^® ^2.0. So the better efficacy on resistant mosquitoes could be impeded either to the higher deltamethrin concentration or to the PBO itself or both. Other field studies did not show an increase of efficacy on resistant *Culex *and pyrethroid susceptible *An. gambiae s.s*. [44] as well as deltamethrin-resistant *Anopheles epiroticus *[45].

### The threat of insecticide resistance mechanisms

This multi-centre study provided also more evidence that pyrethroid resistance can seriously reduce the efficacy of pyrethroid -treated materials in malaria vectors [21,22]. Results obtained in Vallée du Kou showed a strong reduction of ITNs efficacy where the *kdr *mutation frequency was high (e.g. personal protection of CTN washed to just before exhaustion ranged from 88% in Malanville to 24% in Vallée du Kou and the insecticidal effect ranged from 60% in Malanville to 25% in Vallée du Kou). The same trend was observed with PermaNet^® ^2.0, confirming that the *Kdr *mutation is an important predictor of pyrethroid resistance phenotype in malaria vectors as previously described [23,46]. Lower insecticidal activity and personal protection were already demonstrated in West Africa with pyrethroid resistant mosquito populations using either Olyset^® ^net or PermaNet^® ^[47] and also insecticide treated plastic sheetings [48]. Unfortunately, in most malaria endemic countries, *An. gambiae *populations are sharing very high frequency of *Kdr *mutation [8,49-51] alone or in combination with metabolic resistance [16,18]. In Pitoa, where *An. arabiensis *show higher metabolism through elevated oxidase and esterase activity [33], CTN efficacy was intermediate (PP and IE were 63.6% and 33.2%, respectively), suggesting that metabolic resistance could also reduce ITN efficacy [24]. This finding supports the global warning about the spread of the pyrethroid resistance although there is no evidence yet for a malaria control failure using LLIN at an operational scale [52].

## Conclusion

To summarize, the present study showed that the new long-lasting bed nets PermaNet^® ^3.0 caused better efficacy against both *Kdr *and metabolic resistant malaria vectors than PermaNet^® ^2.0. Nevertheless in areas of strong resistance like the Vallée du Kou, a large number of exposed mosquitoes survived after exposure to both LLINs. Then as a short term prospect, it seems essential to evaluate this tool in others areas of strong resistance like southern Benin, southern Nigeria and Côte d'Ivoire. It is also crucial to strengthen the collaboration between companies and Research Institutions to find alternative tools for malaria vector control (e.g. using mixtures of unrelated compounds for LLINs [53-57] and/or the use of insecticide-treated plastic sheeting and LLINs [58]), because the race towards an insecticide with a new mode of action will be long and expensive.

## Competing interests

The authors received financial support from Vestergard Frandsen Company to carry out the experimental huts trials in Burkina Faso and Cameroon. However, the authors have strictly followed the WHOPES procedures for testing and evaluation of the efficacy of Permanet 3.0 against malaria vectors. The Research teams involve in this study (i.e. IRD, CREC, OCEAC, CM and CRA-W) have no competing and commercial interests with the manufacturer.

## Authors' contributions

VC designs the study and drafted the manuscript. JC, RDD, JE, PN carried out bioassays and conducted the experimental hut trials. OP conducted the chemical analysis on nets. MA and JMH helped design the study and critically revised the manuscript. All authors read and approved the final manuscript.

## Supplementary Material

Additional file 1 **Comparison of exophily obtained for free flying wild *Anopheles gambiae *in experimental huts of all countries**. Raw data from the experimental hut trials. Click here for file

Additional file 2 **Comparison of blood feeding rates obtained for free flying wild *Anopheles gambiae *in experimental huts of all countries**. Raw data from the experimental hut trials. Click here for file

Additional file 3 **Comparison of mortality rates obtained for free flying wild *Anopheles gambiae *in experimental huts of all countries**. Raw data from the experimental hut trials. Click here for file

## References

[B1] Snow RW Guerra CA Noor AM Myint HY Hay SI The global distribution of clinical episodes of *Plasmodium falciparum *malaria Nature 2005 434 214 217 10.1038/nature03342 15759000 PMC3128492

[B2] WHO World_Health_Organization World Malaria Report 2008 Geneva: World Health Organization 215

[B3] Lengeler C Insecticide-treated bed nets and curtains for preventing malaria Cochrane Database of Systematic reviews 2009 2 1 58 10.1002/14651858.CD000363.pub2 15106149

[B4] Okell LC Drakeley CJ Bousema T Whitty CJ Ghani AC Modelling the impact of artemisinin combination therapy and long-acting treatments on malaria transmission intensity PLoS Med 2008 5 e226 discussion e226 10.1371/journal.pmed.0050226 19067479 PMC2586356

[B5] Okell LC Drakeley CJ Ghani AC Bousema T Sutherland CJ Reduction of transmission from malaria patients by artemisinin combination therapies: a pooled analysis of six randomized trials Malar J 2008 7 125 10.1186/1475-2875-7-125 18613962 PMC2491628

[B6] Zaim M Aitio A Nakashima N Safety of pyrethroid-treated mosquito nets Med Vet Entomol 2000 14 1 5 10.1046/j.1365-2915.2000.00211.x 10759305

[B7] WHO WHO recommended long-lasting insecticidal mosquito nets http://www.who.int/whopes/Long_lasting_insecticidal_nets_Aug09.pdf

[B8] Chandre F Manguin S Brengues C Dossou Yovo J Darriet F Diabate A Carnevale P Guillet P Current distribution of a pyrethroid resistance gene (kdr) in *Anopheles gambiae *complex from west Africa and further evidence for reproductive isolation of the Mopti form Parassitologia 1999 41 319 322 10697876

[B9] Etang J Fondjo E Chandre F Morlais I Brengues C Nwane P Chouaibou M Ndjemai H Simard F First report of knockdown mutations in the malaria vector *Anopheles gambiae *from Cameroon Am J Trop Med Hyg 2006 74 795 797 16687682

[B10] Stump AD Atieli FK Vulule JM Besansky NJ Dynamics of the pyrethroid knockdown resistance allele in western Kenyan populations of *Anopheles gambiae *in response to insecticide-treated bed net trials Am J Trop Med Hyg 2004 70 591 596 15210997

[B11] Hargreaves K Koekemoer LL Brooke BD Hunt RH Mthembu J Coetzee M *Anopheles funestus *resistant to pyrethroid insecticides in South Africa Med Vet Entomol 2000 14 181 189 10.1046/j.1365-2915.2000.00234.x 10872862

[B12] Hargreaves K Hunt RH Brooke BD Mthembu J Weeto MM Awolola TS Coetzee M *Anopheles arabiensis *and *An. quadriannulatus *resistance to DDT in South Africa Med Vet Entomol 2003 17 417 422 10.1111/j.1365-2915.2003.00460.x 14651656

[B13] Lapied B Pennetier C Apaire-Marchais V Licznar P Corbel V Innovative applications for insect viruses: towards insecticide sensitization Trends Biotechnol 2009 27 190 198 10.1016/j.tibtech.2008.12.005 19251330

[B14] Martinez Torres D Chandre F Williamson MS Darriet F Berge JB Devonshire AL Guillet P Pasteur N Pauron D Molecular characterization of pyrethroid knockdown resistance (kdr) in the major malaria vector *Anopheles gambiae s.s* Insect Mol Biol 1998 7 179 184 10.1046/j.1365-2583.1998.72062.x 9535162

[B15] Ranson H Jensen B Vulule JM Wang X Hemingway J Collins FH Identification of a point mutation in the voltage-gated sodium channel gene of Kenyan *Anopheles gambiae *associated with resistance to DDT and pyrethroids Insect Mol Biol 2000 9 491 497 10.1046/j.1365-2583.2000.00209.x 11029667

[B16] Hemingway J Hawkes NJ McCarroll L Ranson H The molecular basis of insecticide resistance in mosquitoes Insect Biochem Mol Biol 2004 34 653 665 10.1016/j.ibmb.2004.03.018 15242706

[B17] Djouaka RF Bakare AA Coulibaly ON Akogbeto MC Ranson H Hemingway J Strode C Expression of the cytochrome P450s, CYP6P3 and CYP6 M2 are significantly elevated in multiple pyrethroid resistant populations of *Anopheles gambiae s.s*. from Southern Benin and Nigeria BMC Genomics 2008 9 538 10.1186/1471-2164-9-538 19014539 PMC2588609

[B18] Muller P Chouaibou M Pignatelli P Etang J Walker ED Donnelly MJ Simard F Ranson H Pyrethroid tolerance is associated with elevated expression of antioxidants and agricultural practice in *Anopheles arabiensis *sampled from an area of cotton fields in Northern Cameroon Mol Ecol 2008 17 1145 1155 10.1111/j.1365-294X.2007.03617.x 18179425

[B19] Muller P Warr E Stevenson BJ Pignatelli PM Morgan JC Steven A Yawson AE Mitchell SN Ranson H Hemingway J Field-caught permethrin-resistant *Anopheles gambiae *overexpress CYP6P3, a P450 that metabolises pyrethroids PLoS Genet 2008 4 e1000286 10.1371/journal.pgen.1000286 19043575 PMC2583951

[B20] Wondji CS Irving H Morgan J Lobo NF Collins FH Hunt RH Coetzee M Hemingway J Ranson H Two duplicated P450 genes are associated with pyrethroid resistance in *Anopheles funestus *a major malaria vector Genome Res 2009 19 452 459 10.1101/gr.087916.108 19196725 PMC2661802

[B21] Kelly-Hope L Ranson H Hemingway J Lessons from the past: managing insecticide resistance in malaria control and eradication programmes Lancet Infect Dis 2008 DOI:10.1016/S1473-3099(08)70045-8 18374633 10.1016/S1473-3099(08)70045-8

[B22] Corbel V Chandre F Brengues C Akogbeto M Lardeux F Hougard JM Guillet P Dosage-dependent effects of permethrin-treated nets on the behaviour of Anopheles gambiae and the selection of pyrethroid resistance Malar J 2004 3 22 10.1186/1475-2875-3-22 15242513 PMC471558

[B23] N'Guessan R Corbel V Akogbeto M Rowland M Reduced efficacy of insecticide-treated nets and indoor residual spraying for malaria control in pyrethroid resistance area, Benin Emerg Infect Dis 2007 13 199 206 10.3201/eid1302.060631 17479880 PMC2725864

[B24] Etang J Chandre F Guillet P Manga L Reduced bio-efficacy of permethrin EC impregnated bednets against an *Anopheles gambiae *strain with oxidase-based pyrethroid tolerance Malar J 2004 3 46 10.1186/1475-2875-3-46 15569394 PMC538265

[B25] Vulule JM Elevated oxydase and esterase levels associated with permethrin tolerance in *Anopheles gambiae *from Kenyan villages using permethrin-impregnated net Med Vet Entomol 1999 13 239 244 10.1046/j.1365-2915.1999.00177.x 10514048

[B26] Vulule JM Beach RF Atieli FK Roberts JM Mount DL Mwangi RW Reduced susceptibility of *Anopheles gambiae *to permethrin associated with the use of permethrin-impregnated bednets and curtains in Kenya Med Vet Entomol 1994 8 71 75 10.1111/j.1365-2915.1994.tb00389.x 8161849

[B27] Hardstone MC Leichter CA Scott JG Multiplicative interaction between the two major mechanisms of permethrin resistance, kdr and cytochrome P450-monooxygenase detoxification, in mosquitoes J Evol Biol 2009 22 416 423 10.1111/j.1420-9101.2008.01661.x 19196389

[B28] Berticat C Bonnet J Duchon S Agnew P Weill M Corbel V Costs and benefits of multiple resistance to insecticides for *Culex quinquefasciatus *mosquitoes BMC Evol Biol 2008 8 104 10.1186/1471-2148-8-104 18397515 PMC2359736

[B29] WHO WHO_Pesticide_Evaluation_Scheme Report of the twelfth WHOPES working group meeting. WHO/HTM/NTD/WHOPES/20091 2009 Geneva, WHO 1 120

[B30] Bingham G Gunning RV Gorman K Field LM Moores GD Temporal synergism by microencapsulation of piperonyl butoxide and alpha-cypermethrin overcomes insecticide resistance in crop pests Pest Manag Sci 2007 63 276 281 10.1002/ps.1336 17304634

[B31] Corbel V N'Guessan R Brengues C Chandre F Djogbenou L Martin T Akogbeto M Hougard JM Rowland M Multiple insecticide resistance mechanisms in *Anopheles gambiae *and *Culex quinquefasciatus *from Benin, West Africa Acta Trop 2007 101 207 216 10.1016/j.actatropica.2007.01.005 17359927

[B32] Etang J Manga L Chandre F Guillet P Fondjo E Mimpfoundi R Toto JC Fontenille D Insecticide susceptibility status of *Anopheles gambiae s.l*. (Diptera: Culicidae) in the Republic of Cameroon J Med Entomol 2003 40 491 497 10.1603/0022-2585-40.4.491 14680116

[B33] Etang J Manga L Toto JC Guillet P Fondjo E Chandre F Spectrum of metabolic-based resistance to DDT and pyrethroids in *Anopheles gambiae s.l*. populations from Cameroon J Vector Ecol 2007 32 123 133 10.3376/1081-1710(2007)32[123:SOMRTD]2.0.CO;2 17633433

[B34] Dabire KR Diabate A Pare-Toe L Rouamba J Ouari A Fontenille D Baldet T Year to year and seasonal variations in vector bionomics and malaria transmission in a humid savannah village in west Burkina Faso J Vector Ecol 2008 33 70 75 10.3376/1081-1710(2008)33[70:YTYASV]2.0.CO;2 18697309

[B35] Dabire KR Diabate A Namountougou M Toe KH Ouari A Kengne P Bass C Baldet T Distribution of pyrethroid and DDT resistance and the L1014F kdr mutation in *Anopheles gambiae s.l*. from Burkina Faso (West Africa) Trans R Soc Trop Med Hyg 2009 19246066 10.1016/j.trstmh.2009.01.008

[B36] WHO Guidelines for testing mosquito adulticides intended for Indoor Residual Spraying (IRS) and Insecticide Treated Nets (ITNs) 2006

[B37] Scott JA Brogdon WG Collins FH Identification of single specimens of the Anopheles gambiae complex by the polymerase chain reaction Am J Trop Med Hyg 1993 49 520 529 8214283 10.4269/ajtmh.1993.49.520

[B38] Favia G Lanfrancotti A Spanos L Siden Kiamos I Louis C Molecular characterization of ribosomal DNA polymorphisms discriminating among chromosomal forms of *Anopheles gambiae s.s* Insect Mol Biol 2001 10 19 23 10.1046/j.1365-2583.2001.00236.x 11240633

[B39] Pigeon OVJ-P Boinon N Demeulenaere J-L Laduron L Vandecandelaere S Wellin A Herion V Development, validation and performance verification of a new method by GC-FID for the determination of deltamethrin and/or piperonyl butoxide in Long-Lasting (incorporated into polyethylene) Insecticidal Mosquito Nets 2009 CIPAC Technical Meeting San Salvador

[B40] Carnevale P Robert V Anopheles: Biologie, transmission du Plasmodium et lutte antivectorielle 2009 Bondy: IRD

[B41] Gibson G Torr SJ Visual and olfactory responses of haematophagous Diptera to host stimuli Med Vet Entomol 1999 13 2 23 10.1046/j.1365-2915.1999.00163.x 10194745

[B42] Lehane MJ The biology of blood sucking in insects 2005 Cambridge: Cambridge University Press

[B43] Chouaibou M Simard F Chandre F Etang J Darriet F Hougard JM Efficacy of bifenthrin-impregnated bed nets against *Anopheles funestus *and pyrethroid-resistant *Anopheles gambiae *in North Cameroon Malar J 2006 5 77 10.1186/1475-2875-5-77 16961938 PMC1584243

[B44] Tungu P Magesa S Maxwell C Malima R Masue D Sudi W Myamba J Pigeon O Rowland M Evaluation of PermaNet 3.0 a deltamethrin-PBO combination net against *Anopheles gambiae *and pyrethroid resistant *Culex quinquefasciatus *mosquitoes: an experimental hut trial in Tanzania Malar J 2010 9 21 10.1186/1475-2875-9-21 20085631 PMC2817703

[B45] Van Bortel W Chinh VD Berkvens D Speybroeck N Trung HD Coosemans M Impact of insecticide-treated nets on wild pyrethroid resistant *Anopheles epiroticus *population from southern Vietnam tested in experimental huts Malar J 2009 8 248 10.1186/1475-2875-8-248 19874581 PMC2781025

[B46] Donnelly MJ Corbel V Weetman D Wilding CS Williamson MS Black WCt Does kdr genotype predict insecticide-resistance phenotype in mosquitoes? Trends Parasitol 2009 25 213 219 10.1016/j.pt.2009.02.007 19369117

[B47] Dabire RK Diabate A Baldet T Pare-Toe L Guiguemde RT Ouedraogo JB Skovmand O Personal protection of long lasting insecticide-treated nets in areas of *Anopheles gambiae s.s*. resistance to pyrethroids Malar J 2006 5 12 10.1186/1475-2875-5-12 16472385 PMC1402300

[B48] Diabate A Chandre F Rowland M N'Guessan R Duchon S Dabire KR Hougard JM The indoor use of plastic sheeting pre-impregnated with insecticide for control of malaria vectors Trop Med Int Health 2006 11 597 603 10.1111/j.1365-3156.2006.01605.x 16640611

[B49] Chandre F Darriet F Manguin S Brengues C Carnevale P Guillet P Pyrethroid cross resistance spectrum among populations of *Anopheles gambiae s.s*. from Cote d'Ivoire J Am Mosq Control Assoc 1999 15 53 59 10342269

[B50] Diabate A Brengues C Baldet T Dabire KR Hougard JM Akogbeto M Kengne P Simard F Guillet P Hemingway J Chandre F The spread of the Leu-Phe kdr mutation through *Anopheles gambiae *complex in Burkina Faso: genetic introgression and de novo phenomena Trop Med Int Health 2004 9 1267 1273 10.1111/j.1365-3156.2004.01336.x 15598258

[B51] Pinto J Lynd A Vicente JL Santolamazza F Randle NP Gentile G Moreno M Simard F Charlwood JD do Rosario VE Caccone A Della Torre A Donnelly MJ Multiple origins of knockdown resistance mutations in the Afrotropical mosquito vector *Anopheles gambiae* PLoS ONE 2007 2 e1243 10.1371/journal.pone.0001243 18043750 PMC2080755

[B52] Ranson H Abdallah H Badolo A Guelbeogo WM Kerah-Hinzoumbe C Yangalbe-Kalnone E Sagnon N Simard F Coetzee M Insecticide resistance in *Anopheles gambiae*: data from the first year of a multi-country study highlight the extent of the problem Malar J 2009 8 299 10.1186/1475-2875-8-299 20015411 PMC2804687

[B53] Hougard JM Corbel V N'Guessan R Darriet F Chandre F Akogbeto M Baldet T Guillet P Carnevale P Traore-Lamizana M Efficacy of mosquito nets treated with insecticide mixtures or mosaics against insecticide resistant *Anopheles gambiae *and *Culex quinquefasciatus *(Diptera: Culicidae) in Cote d'Ivoire Bull Entomol Res 2003 93 491 498 10.1079/BER2003261 14704095

[B54] Pennetier C Corbel V Boko P Odjo A N'Guessan R Lapied B Hougard JM Synergy between repellents and non-pyrethroid insecticides strongly extends the efficacy of treated nets against *Anopheles gambiae* Malar J 2007 6 38 10.1186/1475-2875-6-38 17394646 PMC1851015

[B55] Pennetier C Corbel V Hougard JM Combination of a non-pyrethroid insecticide and a repellent: a new approach for controlling knockdown-resistant mosquitoes Am J Trop Med Hyg 2005 72 739 744 15964959

[B56] Pennetier C Costantini C Corbel V Licciardi S Dabire RK Lapied B Chandre F Hougard JM Mixture for controlling insecticide-resistant malaria vectors Emerg Infect Dis 2008 14 1707 1714 10.3201/eid1411.071575 18976553 PMC2630727

[B57] Pennetier C Costantini C Corbel V Licciardi S Dabire RK Lapied B Chandre F Hougard JM Synergy between repellents and organophosphates on bed nets: efficacy and behavioural response of natural free-flying *Anopheles gambiae *mosquitoes PLoS ONE 2009 4 e7896 10.1371/journal.pone.0007896 19936249 PMC2775911

[B58] Djenontin A Chabi J Baldet T Irish S Pennetier C Hougard JM Corbel V Akogbeto M Chandre F Managing insecticide resistance in malaria vectors by combining carbamate-treated plastic wall sheeting and pyrethroid-treated bed nets Malar J 2009 8 233 10.1186/1475-2875-8-233 19843332 PMC2776024

