# Supplementary material for: Field efficacy of a new mosaic long-lasting mosquito net (PermaNet® 3.0) against pyrethroid-resistant malaria vectors: a multi centre study in Western and Central Africa
Source: Malar J. 2010 Apr 27;9:113. doi: 10.1186/1475-2875-9-113 (PMC2877060; doi:10.1186/1475-2875-9-113)
Supplement: Additional file 1 — Comparison of exophily obtained for free flying wild Anopheles gambiae in experimental huts of all countries. Raw data from the experimental hut trials. [file 1475-2875-9-113-S1.DOC]

**Additional file 1:**

| **Exophily** | **Sites** | **Results** | **Control** | **Permanet2.0**  **20 washes** | **Permanet3.0**  **20 washes** | **Permanet2.0**  **unwash** | **Permanet3.0**  **unwash** | **CTN**  **Exhaustion** |
| --- | --- | --- | --- | --- | --- | --- | --- | --- |
|  | Malanville  (Benin) | **Total entered** | **285** | **195** | **210** | **243** | **214** | **297** |
|  |  | Deterred,%  IC_95%_ |  | 31.5^a^  [24.8-38.2] | 26.3^a^  [20.2-32.4] | 14.7^a^  [10.2-19.2] | 24.9^a^  [19.0-30.8] | -4.2^a^  [ND] |
|  |  | Exophily,%  IC_95%_ | 42.8^a^  [37.0-48.5] | 60.0^b,c,d^  [53.1-66.8] | 67.6^c^  [61.2-73.9] | 61.3^b,c,d^  [55.1-67.4] | 52.8^d^  [46.1-59.4] | 56.9^b,d^  [51.2-62.5] |
|  |  | Induced exophily,%  IC_95%_ | - | 40.1  [33.1-47.1] | 57.9  [51.1-64.7] | 43.2  [36.8-49.6] | 23.3  [17.5-29.1] | 32.9  [27.4-38.4] |
|  | Pitoa  (Cameroon) | **Total entered** | **401** | **310** | **163** | **105** | **146** | **265** |
|  |  | Deterred,%  IC_95%_ |  | 22.7^a^  [17.9-27.5] | 59.4^a^  [51.7-67.1] | 73.8^b^  [65.2-82.4] | 63.6^b^  [55.6-71.6] | 33.9^a^  [28.1-39.7] |
|  |  | Exophily,%  IC_95%_ | 36.4^a,b^  [31.7-41.1] | 51.9^b^  [46.4-57.5] | 51.5^b^  [43.9-59.2] | 61.9^b^  [52.6-71.2] | 58.2^b^  [50.2-66.2] | 59.6^b^  [53.7-65.5] |
|  |  | Induced exophily,%  IC_95%_ | - | 24.4  [19.5-29.3] | 23.8  [17.1-30.5] | 40.1  [30.5-49.7] | 34.3  [26.4-42.2] | 36.5  [30.6-42.4] |
|  | Vallée du Kou  (Burkina Faso) | **Total entered** | **908** | **788** | **724** | **329** | **463** | **1056** |
|  |  | Deterred,%  IC_95%_ |  | 13.2^a^  [10.8-15.6] | 20.3^a^  [17.3-23.3] | 63.8^b^  [58.5-69.1] | 49.1^c^  [44.5-53.7] | -16.3^d^  [ND] |
|  |  | Exophily,%  IC_95%_  Induced exophily,%  IC_95%_ | 31.7^a^  [28.8-34.8]  - | 67.6^c^  [64.3-70.8]  52.6 [49.0-56.2] | 75.7^b^  [72.4-78.7]  64.3 [60.7-67.9] | 80.5^b^  [75.9-84.5]  71.5 [66.5-76.5] | 79.7^b^  [75.8-83.1]  70.3 [66.1-74.5] | 60.1^c^  [57.2-63.1]  41.6 [38.6-44.6] |

*Values in the same raw sharing a same letter superscript do not differ significantly (P < 0.05)*. ND not determined.
