# Supplementary material for: Field efficacy of a new mosaic long-lasting mosquito net (PermaNet® 3.0) against pyrethroid-resistant malaria vectors: a multi centre study in Western and Central Africa
Source: Malar J. 2010 Apr 27;9:113. doi: 10.1186/1475-2875-9-113 (PMC2877060; doi:10.1186/1475-2875-9-113)
Supplement: Additional file 2 — Comparison of blood feeding rates obtained for free flying wild Anopheles gambiae in experimental huts of all countries. Raw data from the experimental hut trials. [file 1475-2875-9-113-S2.DOC]

**Additional file 2**:

| **Blood**  **feeding** | **Sites** | **Results** | **Control** | **Permanet2.0**  **20 washes** | **Permanet3.0**  **20 washes** | **Permanet2.0**  **unwash** | **Permanet3.0**  **unwash** | **CTN**  **Exhaustion** |
| --- | --- | --- | --- | --- | --- | --- | --- | --- |
|  | Malanville  (Benin) | **Total entered** | **285** | **195** | **210** | **243** | **214** | **297** |
|  |  | Blood fed,%  IC_95%_ | 37.5^a^  [31.9-43.1] | 6.1^b,e^  [2.7-9.5] | 12.6^c^  [8.3-17.3] | 3.7^e^  [1.3-6.0] | 0.4^d^  [-0.4-1.3] | 10.4^b,c^  [6.9-13.9] |
|  |  | Blood feeding  inhibition,% | - | 83.6 | 65.7 | 90.1 | 98.7 | 72.2 |
|  |  | Personal protection,%,(IC_95%_) | - | 88.8 (12)  [84.4-93.2] | 74.8 (27)  [68.9-80.7] | 91.6 (9)  [88.1-95.1] | 99.1 (1)  [97.8-100] | 88.8 (12)  [85.2-92.4] |
|  |  |  |  |  |  |  |  |  |
|  | Pitoa  (Cameroon) | **Total entered** | **401** | **310** | **163** | **105** | **146** | **265** |
|  |  | Blood fed,%  IC_95%_ | 52.1^a^  [47.2-57.0] | 20.3^b,f^  [15.8-24.8] | 24.5^b,c,f^  [17.9-31.2] | 15.2^e,f^  [8.4-22.1] | 28.1^b,d^  [20.8-35.4] | 28.7^c,d^  [23.2-34.1] |
|  |  | Blood feeding  inhibition,% | - | 61.0 | 52.9 | 70.8 | 46.1 | 45.0 |
|  |  | Personal protection,%,(IC_95%_) | - | 69.9 (63)  [64.7-75.1] | 80.9 (40)  [74.7-87.1] | 92.3 (16)  [87.1-97.5] | 80.4 (41)  [73.8-87.0] | 63.6 (76)  [57.7-69.5] |
|  |  |  |  |  |  |  |  |  |
|  | Vallée du Kou  (Burkina Faso) | **Total entered** | **908** | **788** | **724** | **329** | **463** | **1056** |
|  |  | Blood fed,%  IC_95%_ | 75.7^a^  [72.8-78.3] | 48.5^d^  [45.0-52.9] | 36.3^c^  [32.9-39.9] | 35.3^c^  [30.3-40.6] | 20.7^b^  [17.3-24.7] | 49.4^d^  [46.4-52.5] |
|  |  | Blood feeding  inhibition,% | - | 35.9 | 52.0 | 53.4 | 72.6 | 34.7 |
|  |  | Personal protection,%,(IC_95%_) | - | 44.4 (382)  [40.9-47.9] | 61.7 (263)  [58.1-65.3] | 83 (116)  [78.9-87.1] | 86 (96)  [82.8-89.2] | 24 (522)  [21.4-26.6] |

*Values in the same raw sharing a same letter superscript do not differ significantly (P < 0.05)*
