# Supplementary material for: Field efficacy of a new mosaic long-lasting mosquito net (PermaNet® 3.0) against pyrethroid-resistant malaria vectors: a multi centre study in Western and Central Africa
Source: Malar J. 2010 Apr 27;9:113. doi: 10.1186/1475-2875-9-113 (PMC2877060; doi:10.1186/1475-2875-9-113)
Supplement: Additional file 3 — Comparison of mortality rates obtained for free flying wild Anopheles gambiae in experimental huts of all countries. Raw data from the experimental hut trials. [file 1475-2875-9-113-S3.DOC]

**Additional file 3**:

| **Mortality** | **Sites** | **Results** | **Control** | **Permanet2.0**  **20 washes** | **Permanet3.0**  **20 washes** | **Permanet2.0**  **unwash** | **Permanet3.0**  **unwash** | **CTN**  **Exhaustion** |
| --- | --- | --- | --- | --- | --- | --- | --- | --- |
|  | Malanville  (Benin) | **Total entered** | **285** | **195** | **210** | **243** | **214** | **297** |
|  |  | Overall mortality,%  IC_95%_ | 4.2^a^  [1.8-6.5] | 70.7^b,c^  [64.3-77.1] | 70.0^b^  [63.8-76.2] | 88.8^e^  [84.9-92.8] | 96.7^f^  [94.3-99.0] | 61.2^d^  [55.7-66.8] |
|  |  | Insecticidal effect,%  IC_95%_ | - | 69.4  [63.0-75.9] | 68.6  [62.4-74.9] | 88.4  [84.3-92.4] | 96.6  [94.1-99.0] | 59.6  [54.0-65.1] |
|  |  |  |  |  |  |  |  |  |
|  | Pitoa  (Cameroon) | **Total entered** | **401** | **310** | **163** | **105** | **146** | **265** |
|  |  | Overall mortality,%  IC_95%_ | 12.9^a^  [9.7-16.3] | 56.5^b^  [50.9-61.9] | 77.9^c^  [71.5-84.3] | 82.9^c^  [75.6-90.1] | 93.8^e^  [89.9-97.7] | 41.9^d^  [35.9-47.8] |
|  |  | Insecticidal effect,%  IC_95%_ | - | 49.9  [44.4-55.5] | 74.6  [67.9-81.3] | 80.3  [72.7-87.9] | 92.9  [88.8-97.1] | 33.2  [27.6-38.9] |
|  |  |  |  |  |  |  |  |  |
|  | Vallée du kou  (Burkina Faso) | **Total entered** | **908** | **788** | **724** | **329** | **463** | **1056** |
|  |  | Overall mortality,%  IC_95%_ | 4.9^a^  [3.6-6.4] | 30.2^d^  [27.1-33.5] | 49.3^c^  [45.7-52.9] | 44.4^c^  [39.1-49.8] | 78.2^b^  [74.2-81.7] | 28.6^d^  [25.8-31.2] |
|  |  | Insecticidal effect,%  IC_95%_ | - | 26.7  [23.6-29.7] | 46.7  [43.1-50.4] | 41.5  [36.2-46.9] | 77.1  [73.3-80.9] | 24.8  [22.2-27.4] |

*Values in the same raw sharing a same letter superscript do not differ significantly (P < 0.05)*
